# Sudden Death: A Practical Autopsy Approach to Unexplained Mediastinitis Due to Fatal Untreated Neck Infections—A Systematic Review

**DOI:** 10.3390/diagnostics14111150

**Published:** 2024-05-30

**Authors:** Aniello Maiese, Fabio Del Duca, Alessandro Ghamlouch, Biancamaria Treves, Alice Chiara Manetti, Gabriele Napoletano, Alessandra De Matteis, Francesca Dimattia, Huan Wan, Lorenzo Pignataro, Raffaele La Russa

**Affiliations:** 1 Department of Surgical Pathology, Medical, Molecular and Critical Area, Institute of Legal Medicine, University of Pisa, 56126 Pisa, Italy; aniello.maiese@unipi.it; 2 Department of Anatomical, Histological, Forensic and Orthopedic Sciences, Sapienza University of Rome, Viale Regina Elena 336, 00161 Rome, Italy; alessandro.ghamlouch@uniroma1.it (A.G.); biancamaria.treves@uniroma1.it (B.T.); gabriele.napoletano@uniroma1.it (G.N.); 3 Department of Public Health and Infectious Diseases, Sapienza University of Rome, 00161 Rome, Italy; alicechiara.manetti@uniroma1.it (A.C.M.); alessandra.dematteis@uniroma1.it (A.D.M.); 4 Airway Surgery Unit, Department of Surgical Specialties, Bambino Gesù Children’s Hospital, IRCCS, 00165 Rome, Italy; francesca.dimattia@unimi.it; 5 Criminal Justice School, Zhongnan University of Economics and Law, Wuhan 430073, China; wanhuan@stu.zuel.edu.cn; 6 Department of Clinical Sciences and Community Health, University of Milan, 20122 Milan, Italy; lorenzo.pignataro@unimi.it; 7 Otolaryngology Unit, Department of Specialist Surgical Sciences, Fondazione IRCCS Ca’ Granda Ospedale Maggiore Policlinico, 20122 Milan, Italy; 8 Department of Clinical Medicine, Public Health, Life Sciences, and Environmental Sciences, University of L’Aquila, 67100 L’Aquila, Italy; raffaele.larussa@univaq.it

**Keywords:** autopsy, neck surgery, forensic, postmortem, mediastinitis

## Abstract

Neck infections are often prone to being underestimated and can manifest insidiously. The spread of infection can lead to translocation into thoracic areas, causing descending necrotizing mediastinitis (DNM). However, the application of the post-mortem approach in such cases is not well-described in the literature. A literature review was carried out according to the PRISMA methods. Nine papers were included in the final review, revealing different levels of involvement of neck layers that can be linked to different causes. Expertise with respect to the anatomy of the fasciae and spaces of the neck enables an understanding of the pathogenesis of DNM. However, a clear autoptic description was not provided in any of the articles. Therefore, we also employed a practical post-mortem approach to cases of death due to DNM. It is fundamental for pathologists to identify the exact head and neck structures involved. Providing dissectors with support from an otolaryngologist could be useful. This paper could help address such difficult cases.

## 1. Introduction

In a broader context, neck infections can be underestimated because of insidious manifestation. The severity and complexity of such infections may not be readily apparent, leading to difficulties in their correct assessment and timely recognition. Occurrences of abscess expansion are common and lethal. The spread of infection can lead to translocation into thoracic areas, causing mediastinitis. Odontogenic, oropharyngeal, and cervical infections usually develop into necrotizing fasciitis of the neck, extending to soft tissues delimited by the deep cervical fascia. Descending necrotizing mediastinitis is an acute, dangerous infection of the neck and mediastinum, and it is potentially deadly. It usually starts from an oropharyngeal or cervical infection and spreads into spaces and soft tissues of the upper thorax, causing severe impairment of an individual’s general state, including sepsis, shock, and death. An early diagnosis and surgical drainage help to avoid a critical state; however, the reported surgical outcomes have been discouraging, with a mortality rate ranging from 15.5 to 40% [1,2].

Before involving the mediastinum, an infection can disseminate within the muscle fascicles of the neck, thyroid and submandibular glands, and the vascular-nerve bundle of the neck, causing tongue and pharyngeal edema (leading to airway obstruction), descending mediastinitis, pericarditis, necrotizing fasciitis, pleural empyema, and pneumonia [1]. Deep neck infection (DNI) is also associated with thrombosis and the rupture of vessels (the carotid and jugular) due to necrosis and inflammatory processes, sepsis, disseminated intravascular coagulation, and acute respiratory distress syndrome [2]. Symptoms may include fever and swelling of the neck, tongue, and submandibular glands, which, if not promptly treated, can rapidly lead to death via acute mechanical asphyxia [3] or septic shock [4,5].

Post-mortem assessment of the causes of death can be challenging and may have medico-legal consequences. The intricate cervical fascial system complicates dissection in necrotizing purulent neck cases. Indeed, the anatomical structure of the neck is challenging to dissect even in non-pathological settings. The necrotic process can liquefy the fascial layers, leading to confusion among medical examiners.

According to the classic literature, in 1983, Estrera et al. [6] proposed diagnostic criteria for DNM: clinical symptoms of acute infection; typical radiographic findings; documented necrotizing mediastinal infection during surgery; and a relationship between an oropharyngeal or cervical infection and the necrotizing mediastinal process during autopsy. Indeed, a serious debate on conducting neck dissections to find the cause of infection is required.

The aim of this study is to gather scientific knowledge from the literature regarding descending mediastinitis and propose a comprehensive, multidisciplinary approach. This approach has been followed in the case report presented herein. Thanks to the study results and an anatomical approach combining forensic pathology and forensic otolaryngology, we can put forward a proposal for an operational protocol in the case of descending necrotizing mediastinitis. The major causes will be discussed, and their forensic approach will be demonstrated through example cases in non-pathological conditions to promote sector-specific technique awareness.

## 2. Materials and Methods

### 2.1. Eligibility Criteria

The present systematic review was carried out according to the Preferred Reporting Items for Systematic Review (PRISMA) standards [7].

### 2.2. Search Criteria and Critical Appraisal

A systematic literature search and a critical appraisal of the collected studies were conducted. An electronic search of PubMed, Science Direct Scopus, and Google Scholar for articles dating from the inception of these databases to December 2023 was performed. The search terms were (“descending necrotizing mediastinitis” OR “mediastinitis” OR “neck infection” OR “abscess”) AND (“autopsy” OR “postmortem” OR “post mortem” OR “post-mortem”), included in the titles, abstracts, and keywords [all fields]. Bibliographies of all identified documents were reviewed and compared with further relevant literature. Methodological evaluation of each study was conducted according to the PRISMA standards, including assessment of bias.

Papers possessing the following features were included: original research articles, cohort/retrospective studies on forensic and anatomopathological evaluation of sudden death due to mediastinitis, autopsy cases of the mediastinal involvement of neck infections, and submitted and already-published articles, excluding non-published ones; only articles written in English were included.

Meta-analysis, reviews, and systematic reviews were excluded to avoid repetition and data duplication. All data were extracted from suitable articles.

Data collection involved study selection and data extraction. Three researchers (A.M., F.D.D., and P.F.) independently reviewed documents whose titles or abstracts appeared to be relevant and selected those dealing with autopsy cases of descending mediastinitis due to a non-surgically related neck infection. Discordance between the researchers in relation to eligibility was resolved through a consensus process. Unpublished or grey literature was not reviewed. Data extraction was performed by three investigators (A.C.M., A.S., and F.I.) and verified by another investigator (F.D.). Only papers or abstracts written in English were included in the search code.

## 3. Results

**Figure 1:**
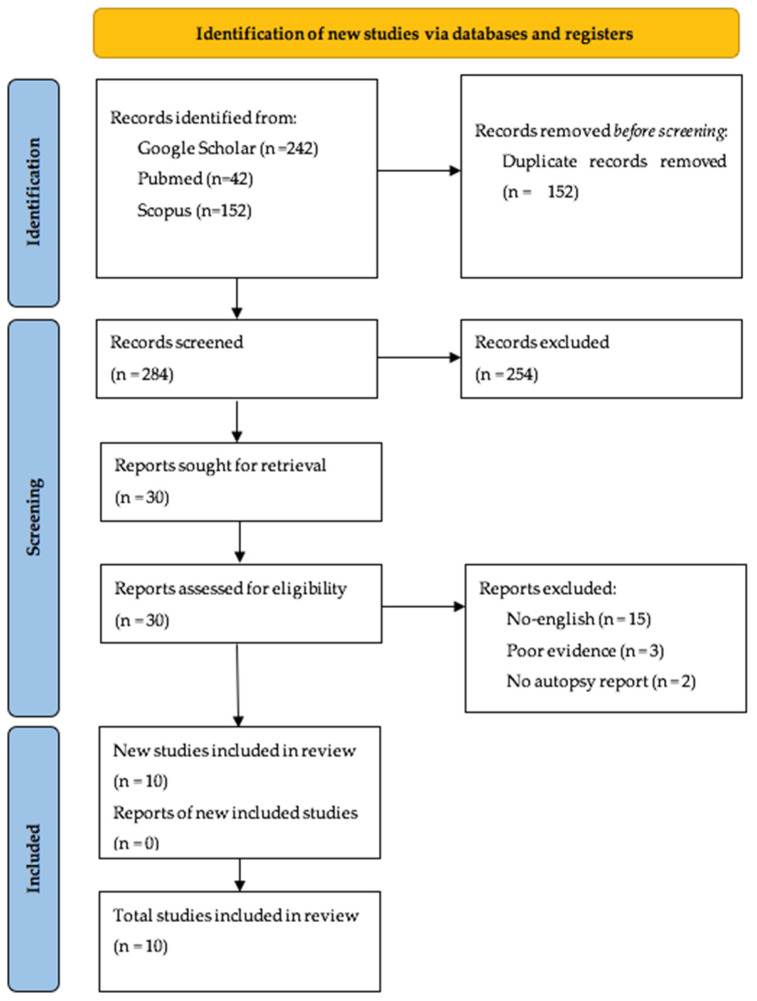
The process of selecting papers according to the PRISMA protocol.

The collected research on autoptic evidence of DNM revealed an absolute predominance of case reports. We found a total of 284 articles, as reported in the PRISMA flowchart, shown in Figure 1. After reading the abstracts, we screened the remaining articles through a critical revision of the entire datasheet (when available).

After the screening was complete, we selected 10 articles using additional criteria, excluding duplicates and papers that did not provide autoptic data. Case reports, research articles, and autopsy series reports were included.

Twelve cases of death related to the mediastinal dissemination of a neck infection were included. Table 1 summarizes the main characteristics of the articles included in this review. The mean age was 41.2 years (Median = 38.5; IQR = 19; IQR_1–3_ = 31.5–50.5; SD = 14.8). As shown, eight out of twelve (66.7%) of the individuals were male, while four (33.3%) were female.

Despite an absence of clear signs of mediastinitis, most of the studies reported clinical presentation (Table 2). In particular, not all the deceased subjects had been hospitalized.

Most of the patients showed symptoms of upper airway infection or masses. In particular, the most important sign was the swelling of the neck and oral cavity, presented by seven out of twelve patients (58.3%). Other symptoms were toothache, present in 33.4% of cases (4/12); fever, present in 25% (3/12); and Ludwig’s Angina, present in 16.7% (2/12). Other minor signs were chest pain, swallowing difficulty, and earache associated with evidence of a hyperemic right tympanum.

Concerning instrumental analysis during hospitalization, the most-reported analyses were computed tomography (CT) scans (4/12) and chest X-rays (3/12). The most-reported signs identified via radiological imaging were evidence of neck cellulitis with emphysema and signs of mediastinitis. X-rays revealed infections of the mediastinum and the involvement of the lungs, whereas a mediastinum shift [12].

The autopsy reports highlighted the widespread involvement of the neck and mediastinum via purulent consolidation, serving the purposes of this study.

In all the cases presented (Table 3), only one case was related to mechanical asphyxia due to an infection of soft tissue that led to neck compression. In most cases, death was attributed to septic shock (92%). To reach a diagnosis, the coroners performed a dissection of the neck, but a clear description of the technique employed for this task was not reported in any of the papers. External examinations were described in only three cases. Miller et al. (2018) [13] observed a greenish discoloration of the body. Musayev et al. (2020) [15], Abbie Tu, Gilbert J.D., and Byard R. (2021) [16] and Bandou et al. (2022) [17] described significant swelling of the neck with local signs of putrefaction, such as greenish discoloration of the neck, subcutaneous emphysema, and evidence of tooth avulsion. In all the cases presented, infective involvement of the mediastinum was found during the autopsy. When purulent collection was carried out, neck dissection was reported in only 4/12 cases. Necrotic and purulent layers underly every neck subfascial area.

## 4. Discussion

Expertise in the anatomy of the neck and knowledge of cervical fasciae and spaces help to clarify the pathogenesis of DNM. Odontogenic, oropharyngeal, and cervical infections often lead to necrotizing fasciitis of the neck, spreading to soft tissues delimited by the deep cervical fascia [18]. The deep cervical fascia is anatomically divided into three layers: the superficial, middle, and deep layers. The superficial layer is a sheet of fibrous tissue around the neck. The middle layer encircles neck muscles and viscera; the part of the middle layer beyond the pharynx is the retropharyngeal layer, also known as the posterior visceral, retro-visceral, retroesophageal, buccopharyngeal, or visceral layer. The deep layer is made up of the alar fascia and prevertebral fascia. The deep cervical fascia layers delimit the virtual spaces of the neck: the retropharyngeal space and danger space [18]. The alar fascia therefore provides an anatomical and functional barrier that hinders the spreading of infections from retropharyngeal spaces into the thorax [18,19]. The upper limit of the alar fascia was identified by Scali et al., who placed it at the C1 vertebral level, while the inferior limit has been set at multiple vertebral levels, ranging from C6 to T2 [20]. The danger space lies posteriorly to the retropharyngeal space, between the soft tissue of the alar fascia anteriorly and the prevertebral fascia posteriorly, and extends directly from the base of the skull through the posterior mediastinum to the diaphragm [21].

There is a hypothetical connection between the subcutaneous and submucosal spaces of the face, the oral cavity, the anterior neck regions, and the superior mediastinum.

The oral cavity connects to the sublingual, submental, and submandibular spaces. The submandibular space is in contiguity with the prestyloid space, followed by the postyloid space. The postyloid space directly connects to the retrovisceral space and danger space, with the latter leading to the superior mediastinum [22].

This is the possible pathway through which an infection spreads from the oral cavity to the superior mediastinum.

An odontogenic origin of infection is present in a percentage of cases ranging from 30% according to Iwata et al. [23] to 76% according to Mora et al. [24], while the presence of peritonsillar abscesses ranges from 0% according to Estrera et al. [6] and 30% according to Freeman et al. [25] to 60.8% according to Roccia et al. [26]. Infections originating from the oral cavity initially extend to the subcutaneous tissue through the superficial deep cervical fascia or in the primary submucous space of the oral cavity, that is, the sublingual space. Spreading further, these infections reach the spaces between the layers of deep cervical fascia and proceed to three potential locations: the pterygomandibular space, the prestyloid space, or the submandibular space. Continuing their spread, these infections then reach the deep fascial spaces. The prestyloid and poststyloid spaces act as relay stations, facilitating the transmission of infections to the superior mediastinum through the retrovisceral space, danger space, pretracheal space, and carotid sheath. The primary causes of deep tissue neck infections are odontogenic infections (38.8–49%), and the latter account for89% of cases of severe multi-space infections. Many factors may be connected with the spreading of odontogenic infections: poor oral hygiene, metabolism, inadequate prevention, or antibiotic therapy [27].

According to Blankson et al., about 40.3% of patients acquire infections related to dental issues, and, among these cases, around 6.2% are affected by dentoalveolar abscesses, while Ludwig’s angina leads to a spreading infection in about 52% of cases. Surgery plays a key role in treating these infections by removing the infection source, if identifiable, and reducing local inflammation through draining pus and removing necrotic tissue. Third molars often trigger acute dental infections, and these may persist even after tooth removal or be linked to surgical or implant procedures. Proper drainage, thorough washing, and the placement of drains are necessary to prevent the accumulation of pus [28].

A peritonsillar abscess is the accumulation of pus between the tonsillar capsule and the pharyngeal constrictor muscle. Its pathogenesis includes acute tonsillitis, and it is likely that the bacteria involved will spread to the peritonsillar space via the salivary duct system [29].

Infections that lead to DNM are mostly polymicrobial and mixed aerobic/anaerobic and affect mainly adults at around their fourth decade. Several authors have studied patients with immune system deficiencies (mainly diabetes), who are more predisposed to developing this disease [26].

A polymicrobial mix of aerobes and anaerobes is commonly analyzed in pus aspirates, but there is evidence suggesting the pathogenic prevalence of Group A Streptococci and Fusobacterium necrophorum [28,29]. Complications are rare and include parapharyngeal abscesses, upper-airway obstruction, Lemierre’s syndrome, necrotizing fasciitis, mediastinitis, erosion of the internal carotid artery, brain abscesses, and streptococcal toxic shock syndrome. The treatment of peritonsillar abscess consists of surgical drainage and antimicrobial therapy. Generally, three methods of drainage are used: needle aspiration, incision, and acute tonsillectomy [30]. So, in the text below, we want to underline the influence of pathologist when using the proper dissection technique. If needed for judicial purposes, it could be useful to involve a microbiologist in the process because identifying the microbiological species could be necessary for public health security or professional litigation.

### 4.1. Diagnosis CT MRI

Early diagnosis and quick intervention are required for head and neck infections. Radiological imaging plays a key role in detecting the location of the disease, its extension, and the source of infection and identifying associated complications. Contrast-enhanced CT is the primary and standard mode of imaging used for these infections because it offers advantages such as immediate availability, cost-effectiveness, and a brief examination time with rapid data acquisition [31]. MRI offers unique benefits compared to CT, including superior contrast resolution, high sensitivity for detecting head and neck abscesses, and less image quality degradation due to metal artifacts associated with dental treatment.

Post-contrast T1-weighted imaging reveals peritonsillar abscesses as localized fluid collections with enhanced margins, while diffusion-weighted imaging shows internal diffusion restriction. In emergency situations, although not typically the primary choice, MRI of the head and neck can offer an accurate assessment of abscess extension and spreading in parapharyngeal and retropharyngeal spaces. In the deep neck region, it is important to perform a diagnosis differentiating cellulitis from an abscess and measure abscess extension. MRI has been reported to outperform CT in visualizing lesions, evaluating extension, and identifying an infection’s origin [32]. The evaluation of mediastinitis in a corpse should be studied through instrumental analysis [33] due to the high resolution of modern MRI.

### 4.2. Autopsy Approach to Infected Neck and DNM

**Figure 2:**
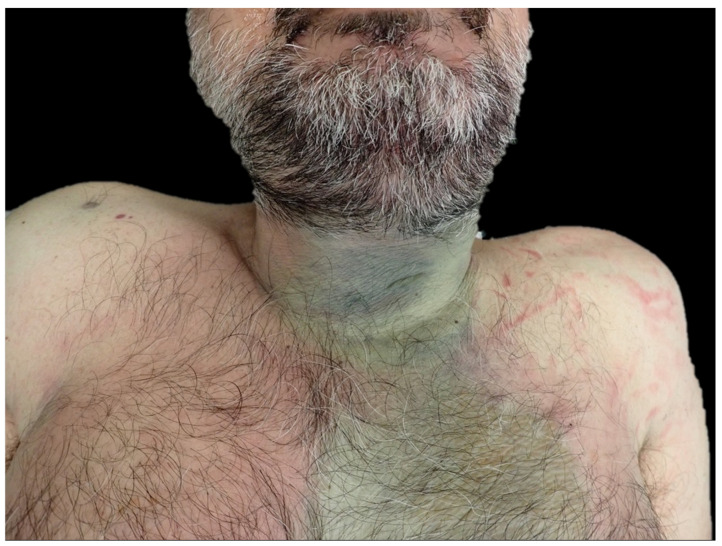
External examination often reveals a green discoloration on the anterior surface of the neck, indicating that putrefactive phenomena are enhanced in this region.

In cases of neck-site infections, post-mortem examination frequently reveals greenish discoloration of the skin. This is due to advanced putrefactive phenomena occurring in the infected tissues. Therefore, in cases of DNM, an external examination often highlights the presence of putrefaction in the neck and anterior surface of the thorax, which are quite rare in common practice. A suspicion of head/neck infection should always arise when such a peculiar putrefaction site is involved (Figure 2), especially if the rest of the body is in a good state of preservation. Another external sign could be skin swelling with or without palpable emphysema.

Dissections should be performed with extreme caution. The first step is to make the classic jugolo-pubic incision useful in both adult and child cases [34]. Putrefaction could alter the thickness and elasticity of the skin; therefore, we suggest carefully flipping the skin layer and the platysma muscle together in order to expose the underlying muscles.

**Figure 3:**
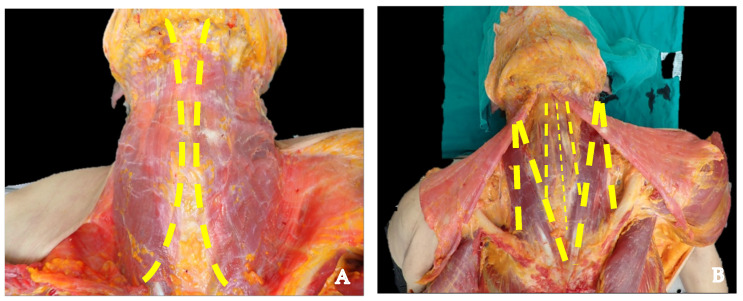
(**A**) Skin dissection approach must be conducted carefully, with platysma exposure. (**B**) Layer-by-layer neck dissection. Yellow: site of dissection.

Personal equipment is required for the adequate protection of health personal in morgues due to physical and biological risks [35]. Then, an each-muscle dissection should be performed (Figure 3), respecting the anatomical layers [36,37]. First, the sternocleidomastoid muscle should be flipped, followed by the infrahyoid muscles, following the anatomical layers (the sternohyoid and omohyoid muscles, serving as the superficial layer, and then the sternothyroid and thyroid muscles).

**Figure 4:**
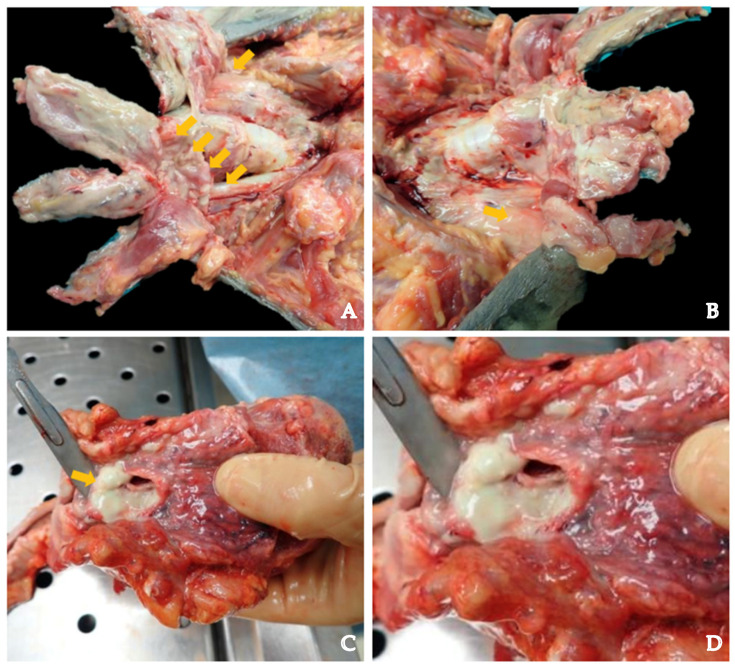
(**A**). The dissection of the neck in a case of DNM. A thick layer of purulent and necrotic material with greenish color is visible on and within the muscle’s fibers of the infrahyoid muscles (yellow arrows). Skin dissection approach must be conducted carefully, with platysma exposure and layer-by-layer neck dissection. (**A**,**B**) shows purulent exudate distribution. (**C**,**D**) show pus surrounding the *aditus ad laringem*.

A dissection should always be conducted starting from the inferior bundle of the muscle. When the thyroid gland is exposed, it should be carefully removed. The pathologist should annotate which neck structures are involved in the infection as the dissection progresses. In case of infection, suppurative material and putrefaction could make it difficult to expose the anatomical structures, so adequate knowledge of normal anatomy is mandatory (Figure 4).

We suggest evaluating whether an infection has spread to the ascendent areas before removing the upper respiratory pathways. A dissection of upper airways begins with cutting the oral floor and pharynx, useful for palate exposure. Blocking the upper airway can help one to evaluate the posterior and middle mediastinum.

If there are signs of ascending spread, our suggestion is to follow the purulent/necrotizing tissue, performing an osteotomy when required. Indeed, in such cases, the removal of the larynx (with or without the tongue) in the traditional way may make it difficult to understand the anatomical relationships between the infected area and the anatomical structures of the neck. After the exposure of the upper infection site, the larynx can be separated from the posterior wall. Our suggestion is to remove it along with the tongue in order to preserve their anatomical relationship. To investigate the presence of infection even in the oral cavity, a palatine bone osteotomy can be performed, unless the pathologist is sure there is no dental involvement.

**Figure 5:**
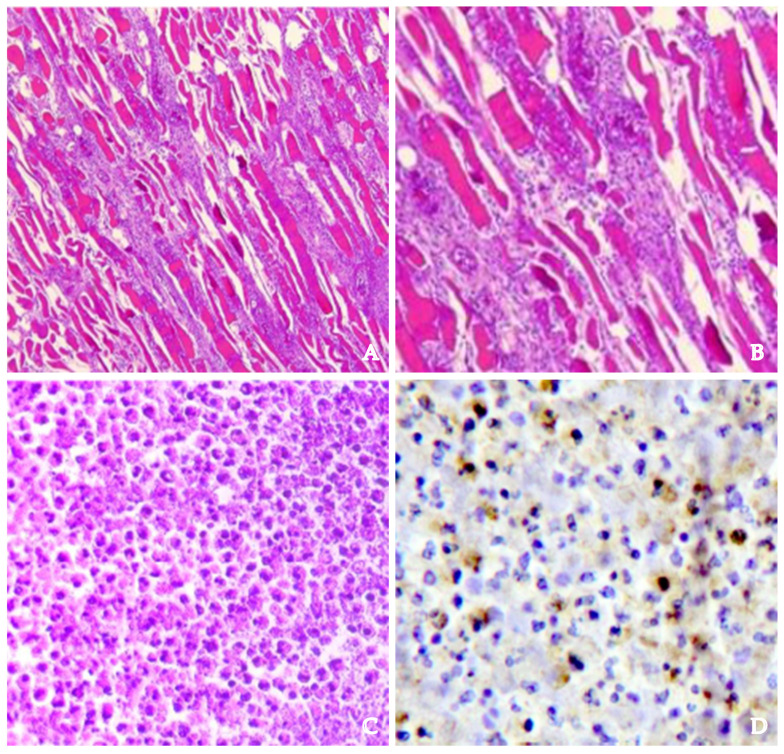
Hematoxylin and eosin stain of infrahyoid muscles (**A**,**B**) show a great degree of infiltration of neutrophils (CD15+). (**C**,**D**) present the same sample with greater magnification (100×).

Then, the pathologist should approach the thorax. After the removal of the sternum and evaluation for the presence of purulent material, a respiratory block should be performed, removing the airways from the tongue to the lungs. Alternatively, the heart could be extracted along with the lungs, partially preserving the anatomical relationship between the mediastinum and the pericardium. After the removal of the thoracic organs, the anterior and posterior thoracic wall should be carefully inspected to verify if the infection has also spread to these structures. In addition to traditional organ samples, histological samples of every layer of soft tissue or gland involved [38] via purulent transformation should be performed. In such cases, purulent tissues may show colliquative necrosis with inflammatory infiltrates, mainly neutrophils and monocytes, alongside fibrinous deposits. Hemorrhagic effusions and leakage of red blood cells in the subendothelial and perivascular spaces may also be present. Abscess-necrotic areas may also indicate the presence of neutrophilic granulocytes (Figure 5). As in inflammation, vessel congestion and microthrombi are other potential histological findings. The involvement of muscle may be proven by detecting features of liquefactive necrosis [39,40,41].

In cases of medical responsibility, it may be important to correlate the descending process with the original infection. Nevertheless, we discourage reliance on bacterial cultures in such cases. It is more reliable to determine the anatomical relationships via macroscopical examination since it is difficult to obtain noncontaminated results in post-mortem swabs [42].

## 5. Conclusions

In cases of DNM, the pathologist needs sound knowledge of the head, neck, and mediastinum anatomy because the infective and later putrefactive artifacts often disrupt the normal tissues relationships.

The data extracted from the systematic review on death due to mediastinitis reveal the important role of circumstance data, which guide the pathologist in researching the sources of infection. We performed a literature review on this topic, collecting ten articles about typical clinical signs and intra-operative approaches.

Based on these data, we conclude that dissector expertise is fundamental for helping a pathologist to identify the exact structures involved. If the dissector has no experience in head (oral cavity) and neck dissection, it could be prudent for them to be supported by an otolaryngologist. The dissection of the neck, even when it was performed in any of the reported cases, was not clearly described in the papers. This is why, in the last section, we propose an essential and practical autopsy approach to infected necks and DNM.

The importance of this article is exemplified by the dangerous nature of neck infections and the possible legal consequences. When DNM occurs, medical litigations could arise. The hypothesis often formed is that a missed or delayed diagnosis or inappropriate therapy is the cause of the dissemination of the infection.

Our paper could provide valid support to clinicians and pathologists when dealing with such intricate cases. MRI and CT premortem show, with great definition, the distribution of necrotizing infections. Further studies could evaluate the role of instrumental analysis (CT or MRI) performed post-mortem.

## Figures and Tables

**Table 1:** The demographic data of the included cases. All reported studies are autopsy case reports/series.

References	Age	Sex
Wenig et al. (1984) [8]	50	Male
Isaacs et al. (1993) [9]	34	Female
Clement et al. (2006) [10]	19	Male
Chatterjee et al. (2014) [11]	29	Male
	40	Male
Shao et al. (2015) [12]	37	Male
Miller et al. (2018) [13]	43	Male
	51	Female
Cascini et al. (2019) [14]	51	Female
Musayev et al. (2020) [15]	37	Male
Abbie Tu, Gilbert J.D., Byard R. (2021) [16]	27	Male
Bandou et al., 2022 [17]	76	Female

**Table 2:** The demographic data of the included cases.

References	Clinical Presentation	Instrumental Analysis
Wenig et al. (1984) [8]	Sore throat [starting at two weeks and steadily worsening], left fucini swelling in the parotid, associated with fever, chills, dysphagia, and mild respiratory distress.	CT scan: The results were positive only for a high degree of subcutaneous emphysema of the anterior chest wall connecting to the left para-pharyngeal space.
Isaacs et al. (1993) [9]	Not reported.	Laryngoscopy: Supraglottic laryngitis with edema of the vallecula, epiglottis, and false vocal cords. CT: Peri-tonsillar cellulitis and edema without evidence of an abscess.
Clement et al. (2006) [10]	N/A	Not performed.
Chatterjee et al. (2014) [11]	Intermittent fever, followed by respiratory distress and hemoptysis.	CT: Homogenous plaque-like soft tissue mass encasing the heart, inseparable from pericardium and walls of cardiac chambers insinuating between the interatrial grooves with a widened interatrial septum.
	Hemoptysis and hematuria.	Chest-X-ray: Moderate cardiomegaly was observed, and echocardiogram revealed minimal pericardial effusion. Echography: Minimal pericardial effusion.
Shao et al. (2015) [12]	Sudden onset of chest tightness and pain 1 h after meal consumption associated with acute respiratory distress (ARD) with massive right hydropneumothorax.	Pre-mortem chest-X-ray: Tight hydropneumothorax with collapsed lung, mediastinum shift to the left, and infiltrates in the left lung
Miller et al. (2018) [13]	Swollen tongue and facial swelling—toothache.	N/A
	Jaw pain and swelling secondary to an abscessed tooth [Ludwig’s angina].	N/A
Cascini et al. (2019) [14]	Earache, angina, and swallowing difficulty. Right tonsillar hypertrophy and a hyperemic right tympanum.	Laryngoscopy: No evidence of intralaryngeal trauma. CT: Air in the posterior mediastinum, which extended from the middle esophagus to the upper neck, and right pleural effusion. Abnormal tissue was noted behind the larynx.
Musayev et al. (2020) [15]	Tooth extraction conducted a month before. Acute onset of fever and swelling of the floor of the mouth.	Premortem: X-ray: signs of inflammation of mediastinum and lungs.
Abbie Tu, Gilbert J.D., Byard R. (2021) [16]	Neck swelling and respiratory distress following a tooth extraction conducted the day before. Surgical incision of the submandibular region, with no release of fluid, was performed. Four hours postoperatively, the patient developed acute respiratory distress, and resuscitation protocol was applied, without success.	Not reported
Bandou et al., 2022 [17]	Sore throat.	

Abbreviation: CT = computed tomography; N/A = Not Applicable.

**Table 3:** A summary of the autopsy and histology results described in the literature.

Reference	External Evaluation	Autopsy	Histology	Cause of Death
Wenig et al. (1984) [8]	N/A	Septic spleen, fatty degeneration of the liver, and thrombosis of the internal jugular vein were observed.		Sepsis related to necrotizing fasciitis.
Isaacs et al. (1993) [9]	N/A	Lungs showed diffuse, organizing alveolar damage, and acute tubular necrosis was evident in the kidneys. Dilatation of the left ventricle, with bacterial thrombotic endocarditis involving the tricuspid, pulmonic, and mitral valves. Postmortem culture of mediastinal tissue-derived gamma Streptococci was conducted.	Multiple thromboemboli in the pulmonary arteries, infarcts of the spleen and thyroid, and ischemic-hypoxic injury of the brain with transtentorial herniation.	Sepsis related to descending necrotizing mediastinitis
Clement et al. (2006) [10]	N/A	The examiners found fibro-purulent effusion in the left pleural cavity associated with mediastinitis.		Oesophageal perforation was the source of empyema, resulting from barotrauma to the lower esophagus caused by vomiting.
Chatterjee et al. (2014) [11]	N/A	Diffuse firm-to-hard infiltrative fibrous lesion was identified involving the middle mediastinum encasing the heart (700 g) with pericardium and the surrounding lung parenchyma. The parietal pericardium was markedly thickened by fibrosis, which encased the pulmonary artery, aorta, and its branches and the medial surface of pleurae.	Fungal profiles with numerous septae (*Aspergillus*) and dense chronic inflammatory infiltrate including many eosinophils.	Acute heart failure due to mediastinal mass from *aspergillus* infection.
	N/A	Mediastinum solidified with firm-to-hard white mass, involving both atria, especially the left atrium, pericardium, the roots of aorta and pulmonary artery, superior vena cava, and hilar region of the lungs, more so with respect to the left lung adjoining left atrium. of the heart.	Aspergillus granulomas involving all chambers.	
Shao et al. (2015) [12]	N/A	Food material in the right pleural space. Evidence of a longitudinal esophageal rupture measuring 5 cm just above the junction of the aortic arch.	Not reported	Mediastinitis secondary to a spontaneous esophageal rupture
Miller et al. (2018) [13]	Phase of decomposition, with bloating of the face, abdomen, and scrotum.	The subcutaneous and subgaleal tissues of the right scalp were edematous, and the right sternocleidomastoid muscle (SCM) showed green–brown discoloration and softening.	Right SCM/Tongue/epiglottis/adventitia of trachea—acute inflammation; left anterior descending coronary artery–thrombus and local inflammation.	complications of submandibular space infection, with other significant conditions contributing to the patient’s death being noted as “atherosclerotic and hypertensive cardiovascular disease”
		The left-cheek mucosa and gingiva of the left side of the mandible were edematous, with necrotic tissue and purulent fluid. Purulent fluid and necrosis of the anterior musculature and fascial tissues bilaterally and extending into the anterior mediastinum were observed. The epicardial surface displayed green discoloration with fibro-purulent adhesions.	Gingival tissue and neck musculature—acute and chronic inflammation with necrosis and granulation tissue. Heart—bacterial overgrowth along the epicardial surface, and perivascular and interstitial fibrosis.	Sepsis due to an abscessed tooth
Cascini et al. (2019) [14]	N/A	Dissection of the neck: purulent necrotizing collection behind the esophagus, connected to a fracture of the right superior horn of the thyroid cartilage.	Mediastinal and retropharyngeal soft tissues—inflammatory accumulation with neutrophils, food residues, and epithelial cells from the oral cavity. Pharyngeal mucosal—ulcerated with fibrin deposition and signs of microperforation caused by the fracture with sharp edges of the right superior horn of the thyroid cartilage. Thyroid cartilage—signs of vital reaction were detected, as massive inflammatory reaction with neutrophil infiltration surrounded the lesion of the thyroid cartilage. Lungs—stasis and an inflammatory response around foreign cells coming from upper airway.	Septic shock via mediastinitis due to an undetected thyroid cartilage fracture secondary to physical assault.
Musayev et al. (2020) [15]	Swelling in the submandibular space and cervical region, and crepitation during palpation.	The 34th tooth space revealed a dry socket associated with hyperemia and swelling. Margins at the floor of the mouth were hyperemic and edematous. Accumulation of purulent exudate at the floor of the mouth and partly inside the mouth was observed. Fibro-purulent collection spread among soft tissue and skeletal muscles, from mouth floor to mediastinum and pleural surface. The examiners noted abundant lymphocyte and neutrophil infiltration.	Soft tissues around the 34th tooth socket—edema, hyperemia, granulation tissue formation, abundant lymphocyte and neutrophil infiltration, and micro-abscess formation. Soft tissue and skeletal muscles of the neck region—areas of necrosis and edema.	Ludwig’s angina complicated by mediastinitis and aspiration pneumonia due to extraction of the 34th tooth.
Abbie Tu, Gilbert J.D., Byard R. (2021) [16]	Well-nourished adult white male (height, 183 cm; weight, 82 kg; body mass index, 24.5). Natural teeth, with a recent extraction of the right lower first molar.	Generalized edema of the neck soft tissues with mild interstitial hemorrhage in the right digastric muscle, around the right submandibular gland, with an increase in the size of the cervical lymph nodes. Marked bilateral submucosal edema of the epiglottis, glottic inlet, and tonsils sufficient to cause airway obstruction was observed.	Submandibular space samples: diffuse cellulitis characterized by edema and neutrophil infiltrates within connective tissue, sparing salivary glands and other glandular structures. Focal micro-abscess formation in glands areas. Some inflammatory involvement of skeletal muscle was present, particularly involving the right digastric muscle. Marked submucosal edema with diffuse neutrophilic infiltration was noted in sections from the glottic inlet.	Acute Asphyxia.
Bandou et al. 2022 [17]	A large blue-green discoloration was found on the right cheek, and the right side of her face was swollen	White pus surrounding the subcutaneous region of the right cheek and the anterior neck, the right sternohyoid muscle, and the region spanning from the pharynx to the dorsal surface of the larynx and esophagus [posterior pharyngeal gap]. Mediastinal abscesses, pleuritis, and pericarditis were observed. There was no apparent airway obstruction. All the teeth were unstable, gingival recession and gingival redness were present, and the hygienic conditions of the individual were quite poor.	Kidney: microthrombi within the glomeruli. Spleen: neutrophil colonies.	Septic shock caused by periodontal disease.

## Data Availability

The data presented in this study are available on request from the corresponding author.
